# Characteristics and Cytological Analysis of Several Novel Allopolyploids and Aneuploids between *Brassica oleracea* and *Raphanus sativus*

**DOI:** 10.3390/ijms25158368

**Published:** 2024-07-31

**Authors:** Mingyang Hu, Shiting Fang, Bo Wei, Qi Hu, Mengxian Cai, Tuo Zeng, Lei Gu, Hongcheng Wang, Xuye Du, Bin Zhu, Jing Ou

**Affiliations:** 1 School of Life Sciences, Guizhou Normal University, Guiyang 550025, China; 222100100386@gznu.edu.cn (M.H.); 232100100391@gznu.edu.cn (S.F.); 232100100408@gznu.edu.cn (B.W.); 232200101543@gznu.edu.cn (Q.H.); 201706002@gznu.edu.cn (M.C.); zengtuo@gznu.edu.cn (T.Z.); leigu1216@gznu.edu.cn (L.G.); wanghc@gznu.edu.cn (H.W.); duxuye@gznu.edu.cn (X.D.); 2 College of Forestry, Guizhou University, Guiyang 550025, China

**Keywords:** interspecific hybridization, genome, fluorescence in situ hybridization, homoeologous chromosome pairing, unreduced single genome

## Abstract

Polyploids are essential in plant evolution and species formation, providing a rich genetic reservoir and increasing species diversity. Complex polyploids with higher ploidy levels often have a dosage effect on the phenotype, which can be highly detrimental to gametes, making them rare. In this study, offspring plants resulting from an autoallotetraploid (RRRC) derived from the interspecific hybridization between allotetraploid *Raphanobrassica* (RRCC, 2n = 36) and diploid radish (RR, 2n = 18) were obtained. Fluorescence in situ hybridization (FISH) using C-genome-specific repeats as probes revealed two main genome configurations in these offspring plants: RRRCC (2n = 43, 44, 45) and RRRRCC (2n = 54, 55), showing more complex genome configurations and higher ploidy levels compared to the parental plants. These offspring plants exhibited extensive variation in phenotypic characteristics, including leaf type and flower type and color, as well as seed and pollen fertility. Analysis of chromosome behavior showed that homoeologous chromosome pairing events are widely observed at the diakinesis stage in the pollen mother cells (PMCs) of these allopolyploids, with a range of 58.73% to 78.33%. Moreover, the unreduced C subgenome at meiosis anaphase II in PMCs was observed, which provides compelling evidence for the formation of complex allopolyploid offspring. These complex allopolyploids serve as valuable genetic resources for further analysis and contribute to our understanding of the mechanisms underlying the formation of complex allopolyploids.

## 1. Introduction

Polyploidy, also known as whole-genome duplication events, results in the duplication of all genes in the genome, providing the original genetic material for biological evolution and acting as an accelerator of evolutionary processes [1,2]. The doubling of chromosomes in the nucleus following polyploidy, along with their inheritable manner in heritance, allows offspring to acquire valuable genes from their parents, enhancing their adaptability to the environment [3,4,5,6]. Polyploidy, especially allopolyploidy, plays a significant role in plant formation, which explains the widespread presence of polyploid plants in nature, such as upland cotton (*Gossypium hirsutum*, AADD, 2n = 52), wheat (*Triticum aestivum*, AABBDD, 2n = 42), *Brassica napus* (AACC, 2n = 38), etc. [7]. Previous studies have noted a particularly high frequency of polyploidy in the evolution of flowering plants, with doubling events occurring before the differentiation of existing angiosperms and seed plants, potentially contributing significantly to the development of flowers and seeds [8]. Research on plant polyploidy holds both theoretical significance and practical application value for biological evolution, species conservation, and genetic breeding [9]. Some researchers have proposed the dosage effect theory, suggesting that changes in an organism’s gene copy number lead to corresponding modifications in phenotype. 

Polyploids can be classified into two main types: autopolyploids and allopolyploids based on the origin of the chromosome set [10,11]. Autopolyploids arise from an increase in ploidy within a single species, often occurring within an individual. In contrast, allopolyploids are formed through hybridization between two different species, each contributing a complete set of chromosomes to the offspring, after doubling the genome [12]. In nature, allopolyploids are more common than autopolyploids, partly due to their more regular meiotic segregation, which enhances normal fertility. Additionally, a complex form of polyploidy, known as autoallopolyploidy, has been observed in certain plant species, including *Triticum macha* [13], perennial sunflower (*Helianthus tuberosus*) [14], and forage grass timothy (*Phleum pratense*) [15], either naturally or through artificial synthesis [16,17]. However, these complex polyploidies remain rare due to irregular chromosome pairing and segregation, and there is limited knowledge about the progenies of these autoallopolyploids. 

Meiosis is a unique form of cell division that occurs during the formation of germ cells in sexually reproductive individuals, distinguishing it from mitosis and amitosis [18]. During meiosis, genetic recombination between non-sister chromatids of homologous chromosomes enhances the genetic diversity of gametes, thereby increasing the adaptability of offspring to their environment [19]. Furthermore, the pairing of homoeologous chromosomes plays a crucial role in demonstrating kinship, as it is a prerequisite for ensuring introgression between parental genomes. Knowledge of the frequency of chromosome pairing not only provides insight into the stability of the genomes but also predicts the degree of genetic diversity of gametes. Therefore, focusing on the meiotic process in pollen mother cells can enhance our cytogenetic knowledge of plants and provide essential cytogenetic information for various fields, such as developmental biology, crossbreeding, and gene localization.

Brassicaceae, a large family of angiosperms, consists of 338 genera and over 3500 species, including many economically valuable crops [20,21]. The genus *Brassica*, the most crucial genus within this family, encompasses a variety of vegetables, flavorings, and oil crops, contributing to around 12% of the world’s edible vegetable oil production. The ‘triangle of U’ theory has been proposed to explain the evolution and interrelationships among different *Brassica* species, serving as an ideal model for studying polyploidy in *Brassica* and other plants. The triangle of U consists of six *Brassica* species, including three diploids: *B. rapa* (2n = 20, AA), *B. oleracea* (2n = 18, CC), and *B. nigra* (2n = 16, BB), as well as three allotetraploids that resulted from continuous selection and evolution: *B. napus* (2n = 38, AACC), *B. juncea* (2n = 36, AABB), and *B. carinata* (2n = 34, BBCC) [22]. Among these, *B. oleracea* is one of the most widely cultivated vegetables worldwide and ranks among the most significant vegetable types in many countries. The portion consumed by humans is actually a manifestation of cabbage’s morphological variation, which is abundant and diverse [23]. 

*Raphanus sativus* (RR, 2n = 18)*,* commonly known as radish, is a biennial or annual herb characterized by its edible fresh roots and holds significant economic importance within the Brassicaceae family [24]. Furthermore, radish demonstrates a high degree of resistance to various diseases that frequently result in substantial yield reductions in Brassica vegetable crops [24,25,26,27,28,29]. Phylogenetic analyses exhibited that *R. sativus* and *Brassica* species shared a very close phylogenetic relationship [30], leading to the successful creation of novel materials that enhance tolerance to both abiotic and biotic stresses in *Brassica* crops, such as beet cyst nematode and clubroot diseases, through crossbreeding with radish [31,32]. Consequently, interspecific hybridizations targeting the development of novel allopolyploids or the conveyance of advantageous characteristics between *Brassica* species and radish have been conducted for roughly two centuries. In addition, *Raphanobrassica*, which is derived from the interspecific hybridization between radish and *B. oleracea,* serves as the first and classical example of artificial allopolyploidy [33], opening an avenue for allopolyploid analysis. 

In a previous study, a *Raphanobrassica* line was reconstructed by combining maternal radish and paternal *B. oleracea* [34]. Furthermore, several unexpected backcrossing progenies with the genome configurations of RRRC (2n = 36) and CCCR (2n = 36) were obtained from reciprocal crosses between *Raphanobrassica* and its diploid parents [35]. It is important to explore how these unexpected hybrids are generated and what types of offspring they produce. However, offspring plants were only obtained from the hybrid with a genome configuration of RRRC under the artificial pollination and embryo rescue technology. The observed genotypes of these progeny were either euploid or nearly euploid, with a higher degree of complexity and polyploidy in their chromosome composition. Our investigation revealed the presence of unreduced single C genome chromosomes, which we hypothesized could be responsible for the formation of these complex polyploids. The existence of these autoallopolyploids could significantly impact future hybridization with Brassicaceae crops, potentially leading to the creation of new species and expanding the genetic diversity of cruciferous crops.

## 2. Results

### 2.1. Determination of Genome Configuration of These Offspring Plants of RRRC

In this study, only 20 embryos were obtained from the parental RRRC plant through artificial supplementary pollination (Table 1). Out of these, 16 offspring plants were successfully germinated with embryo rescue. Analysis of chromosome numbers in premetaphase mitotic cells revealed that seven offspring plants have chromosome sets of 45 (five plants) and 54 (one plant), with variations in ploidy levels of the basic C/R genome. The remaining plants (ten in total) showed alterations in chromosome numbers, differing by one or two chromosomes from the mentioned ploidies.

**Figure 1:**
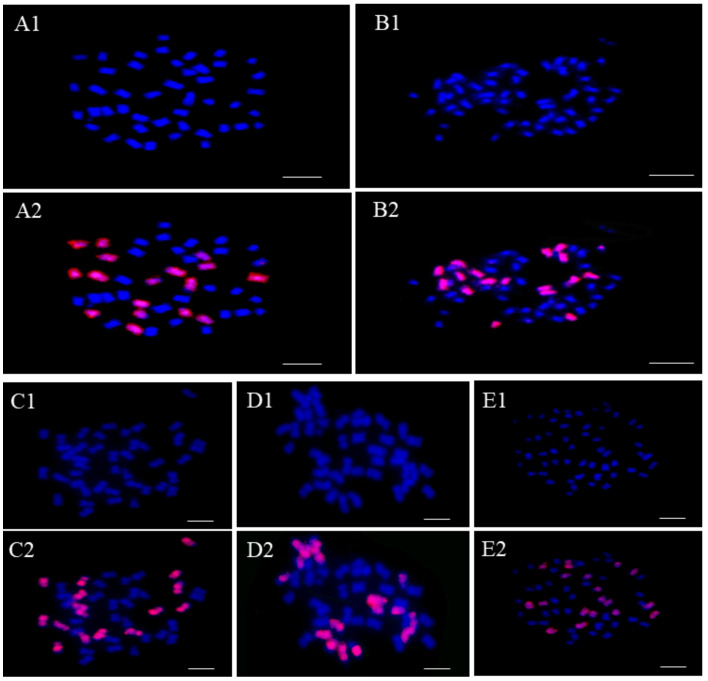
FISH analyses of chromosome constitutions of the offspring plants. Blue signals indicate DAPI staining, and red signals labeled by BAC BoB014O06 probe indicate C subgenome chromosomes. (**A1**,**A2**) The mitotic cell of RRRCC with 27R genome chromosomes (blue) and 18C genome chromosomes (red). (**B1**,**B2**) The mitotic cell of RRRRCC with 36R genome chromosomes (blue) and 18C genome chromosomes (red). (**C1**,**C2**) The mitotic cell of RRRCC^2^ with 27R genome chromosomes (blue) and 16C genome chromosomes (red). (**D1**,**D2**) The mitotic cell of RRRCC^1^ with 27R genome chromosomes (blue) and 17C genome chromosomes (red). (**E1**,**E2**) The mitotic cell of RRRRCC^1^ with 36R genome chromosomes (blue) and 19C genome chromosomes (red). Bar: 10 μm.

To further analyze the genome configuration, FISH (Figure 1) was employed using C-genome-specific repeats as probes (red signals). The plants with a chromosome set of 2n = 45 exhibited 18 red signals and 27 blue signals, representing an RRRCC chromosome configuration (Figure 1(A1,A2)). Similarly, the plant with a chromosome set of 2n = 54 showed 18 red signals, indicating a genome composition of RRRRCC (Figure 1(B1,B2)). Four plants with a chromosome number of 2n = 43 displayed 16 red signals and 27 blue signals, denoted as RRRCC^2^ (Figure 1(C1,C2)). Five progenies with 2n = 44 showed 16 red signals and 27 blue signals, signifying the absence of one C chromosome, labeled as RRRCC^1^ (Figure 1(D1,D2)). Lastly, the plant with 2n = 55 showed 36 R chromosomes and 19 red signals (Figure 1(E1,E2)), indicating a genome composition of RRRRCC^1^.

### 2.2. Morphological Characteristics and Pollen Fertility of Offspring Plants

During the flowering period, detailed examinations and photographs were taken of the offspring to document their reproductive organs, pollen fertility, and leaf characteristics. These offspring plants with distinct genotypes displayed noticeable variations at different growth stages and showed traits that leaned more toward one parent to varying degrees. The majority of these plants exhibit slower growth and later flowering compared to *B. oleracea*. Based on their specific morphological features, these offspring appeared distinguishable.

**Figure 2:**
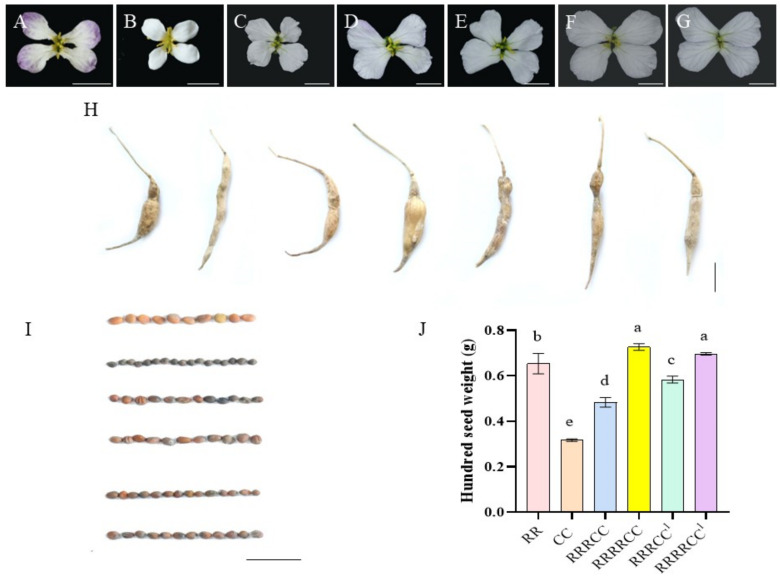
Characteristics of reproductive organ types of *R. sativus*, *B. oleracea*, and these offspring of RRRC. (**A**–**G**) The morphology of flowers of *R. sativus*, *B. oleracea,* RRRCC, RRRRCC, RRRCC^2^, RRRCC^1^, and RRRRCC^1^, respectively. The flower edges of RRRCC^1^ and RRRRCC^1^ are tinged with pale purple. (**H**) Siliques traits of *R. sativus*, *B. oleracea,* and these offspring plants. (**I**,**J**) Seeds traits of *R. sativus*, *B. oleracea*, RRRCC, RRRRCC, RRRCC^1^, and RRRRCC^1^, respectively. Bar: 1 cm. Shared letters a–e are significantly different detected by one-way analysis of variance (ANOVA), *p* < 0.05.

**Figure 3:**
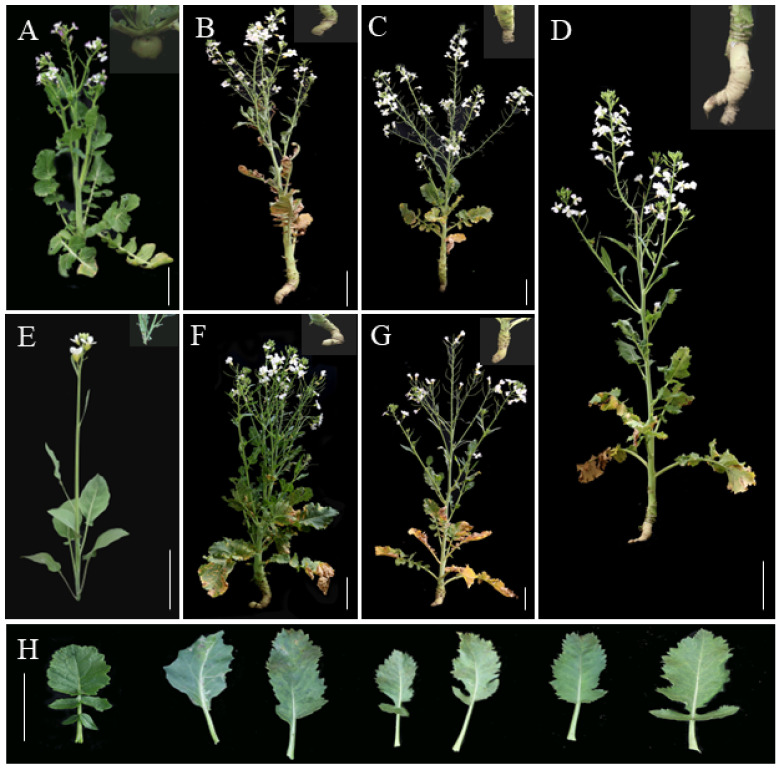
The integrated agronomic traits of *R. sativus*, *B. oleracea,* and the offspring of RRRC. (**A**–**E**) Phenotype of *R. sativus*, *B. oleracea,* RRRCC^2^, RRRCC^1^, RRRCC, RRRRCC, and RRRRCC^1^, respectively. (**H**) Leaves of *R. sativus*, *B. oleracea*, and the offspring of RRRC. Bar: 10 cm.

The RRRCC plants exhibited a preference for *B. oleracea* with white petals (Figure 2C), while their siliques and seeds were more similar to *R. sativus* (Figure 2H,I) with seed weight (0.48 ± 0.02 g) falling between those of the parental plants (Figure 2). Although their roots did not swell as much as those of the parental radish, their high lignification supported robust root development (Figure 3D). The petals of the RRRRCC plant were white with faint purple hues along the edges (Figure 2D), and their siliques were shorter and thicker, resembling radish. In contrast, those of RRRRCC^1^ were longer and thinner, more similar to *B. oleracea* but with a long beak at the top. It was noted that an increase in the R chromosome led to heavier seeds, while an increase in the C chromosome resulted in lighter seed weight. Specifically, the seed weight of RRRRCC (0.73 ± 0.02 g) and RRRRCC^1^ (0.70 ± 0.01 g) was heavier than that of other offspring and parents, with the latter being slightly lighter than the former. Similarly, the seed weight of RRRCC was lighter than that of RRRCC^1^ (0.58 ± 0.015 g) but heavier than those of *B. oleracea* (0.32 ± 0.02 g) (Tukey’s HSD, *p* < 0.05). However, RRRCC^2^ yielded very few seeds and could not be used for statistical purposes. This trend was also reflected in the number of branches, as RRRRCC had more branches compared to RRRCC, but its root did not show significant enlargement (Figure 3F).

The petals of RRRCC^2^ (Figure 2E) and RRRCC^1^ (Figure 2F) were both white, distinguishing them from other plants with dark veins on the petals. RRRCC^1^ had a less prominent root compared to its stem, allowing for clear differentiation between the two plants (Figure 3B,C). RRRRCC^1^ shared similarities with RRRRCC but had fewer branches (Figure 2H and Figure 3G). The floral organs in these progenies (Figure 2C–H) developed in a specific sequence, with ovary emergence preceding stamen growth. These progenies had dentate leaf edges and lanceolate shapes (Figure 3H). In contrast to other offspring, RRRCC^1^, RRRCC, and RRRRCC^1^ had compound leaves, although not as abundant as in radish. The posterior region of the radish leaf was hairy in both seedling and adult stages, while the offspring with smooth leaves lacked this feature in both stages, resembling *B. oleracea* instead. In general, these hybrids exhibited a larger plant architecture compared to the two parent plants. 

**Figure 4:**
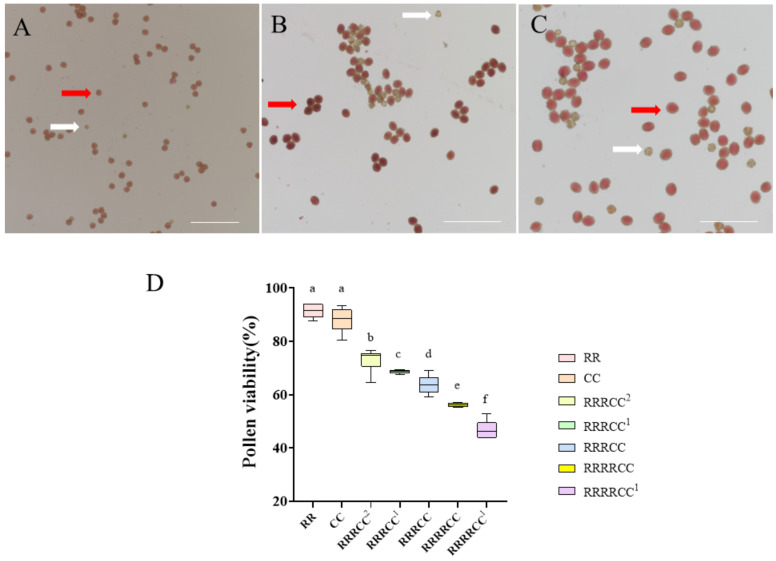
Pollen fertility analysis of *R. sativus*, *B. oleracea*, and the offspring of RRRC. (**A**–**C**) The pollen characteristics of *R. sativus*, RRRCC, and RRRRCC. The deep brown pollens indicate fertility, while the light brown ones indicate abnormal fertility. The red solid arrows indicate fertile pollen grains, and white solid arrows indicate sterile pollen grains. Bar: 100 µm. (**D**) Comparison of pollen fertility across parental species and above-mentioned hybrids. Shared letters a–f are extremely significantly different as detected by one-way analysis of variance (ANOVA), *p* < 0.05.

Pollen fertility analysis of offspring plants was conducted during the flowering stage, with each plant having more than three flowers (Figure 4). Six images were captured per flower using an Olympus microscope equipped with a CCD camera. Subsequently, a one-way analysis of variance (ANOVA) was performed to assess the significance between them. To enhance the reliability of the data, pollen grains from a sample size ranging from 947 to 1249 were considered for the calculation of pollen fertility statistics, corresponding to each tested hybrid and its parent plant. Based on the statistical analysis, the pollen grain germination rates for these hybrids and their parent lines ranged from 467 to 948. As depicted in Figure 4, darker-hued pollens indicated fertility, while lighter-colored ones lacked fertility. The results revealed that the fertility of these offspring plants ranging from 46.88% to 73.06% was notably lower than that of radish (88.00 ± 2.93%) and *B. oleracea* (88.00 ± 2.93%) (Tukey’s HSD, *p* < 0.05). These euploid RRRCC plants exhibited a higher fertility rate (63.79% ± 3.52%) than the RRRRCC plant (56.15% ± 0.71%). Among the offspring, the aneuploid RRRCC^2^ displayed the highest level of fertility (73.06 ± 4.37%), while RRRRCC^1^ showed the lowest level of fertility (46.88 ± 3.43%). In summary, the fertility of these offspring plants decreased as the number of R/C chromosomes increased. Also, more than 900 pollen grains per offspring were included in the statistics. Overall, the pollen germination rate of each progeny was lower than pollen fertility (Figure S1). And with the increase in the R genome chromosome and C genome chromosome, the pollen germination rate decreased. The pollen germination rate of RRRCC^2^ was the highest, while that of RRRRCC^1^ was the lowest.

### 2.3. Chromosome Pairing Patterns of Offspring Plants 

**Figure 5:**
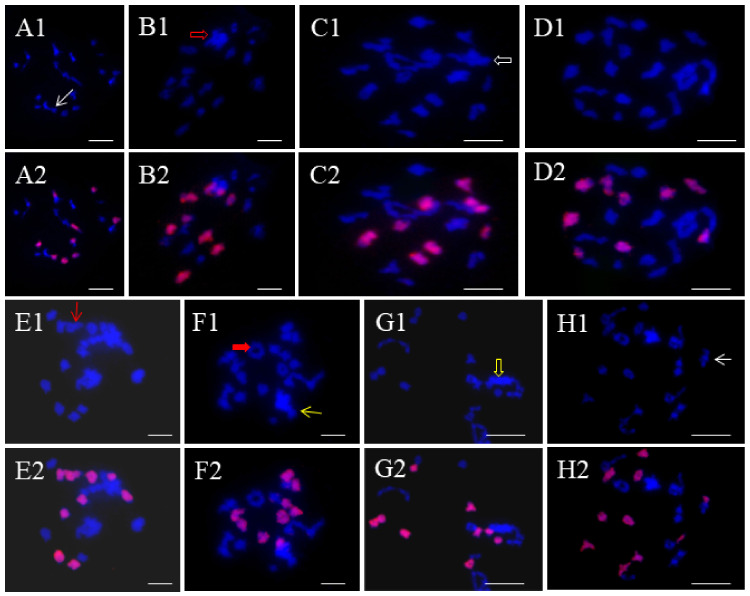
BAC-FISH analyses of chromosome behaviors of the offspring of RRRC at diakinesis. Blue signals indicate DAPI staining, and red signals from BAC BoB014O06 probe indicate C subgenome chromosomes. (**A1**,**A2**) Pollen mother cell (PMC) chromosomal configurations of RRRCC^2^ with a C-R-R trivalent (white arrow). (**B1**,**B2**) PMC chromosomal configurations of RRRCC^1^ with seven C-C bivalents and a C-C-R-R-R-C hexavalent (red hollow arrow). (**C1**,**C2**) PMC chromosomal configuration of RRRCC. One diakinesis with seven C-C bivalents and a C-C-C-C-R-R hexavalent (white hollow arrow). (**D1**,**D2**) PMC chromosomal configurations of RRRCC with six C-C bivalents, a C-C-C trivalent, a univalent, and a C-C-R-R quadrivalent. (**E1**,**E2**) PMC chromosomal configurations of RRRRCC. One diakinesis with seven C-C bivalents and a C-C-C-C-R pentavalent (red arrow). One diakinesis with seven C-C bivalents, a C-C-C trivalent, and a C-R-R-R-R-R hexavalent (yellow arrow). (**F1**,**F2**) An apparent R-R-R-R quadrivalent seems to be present (red solid arrow). PMC chromosomal configurations of RRRRCC^1^. (**G1**,**G2**) One diakinesis with seven C-C bivalents, a C-C-C trivalent, and a C-C-R-R-R-R hexavalent (yellow solid arrow). (**H1**,**H2**) One diakinesis with seven C-C bivalents, a C-C-C trivalent, a univalent, and a C-R-R trivalent (white arrow). Bar, 10 μm.

FISH analysis was conducted using a C-genome-specific probe to investigate chromosome pairing patterns in offspring plants (Figure 5). Each plant had 55 to 66 PMCs at diakinesis, with irregular chromosome pairings like univalent, trivalent, and quadrivalent observed (Table 2). Homoeologous chromosome pairing (HEP) was prevalent in all offspring plants, with RRRRCC^1^ showing the highest incidence of HEP at 78.33% of 60 PMCs (Tukey’s HSD, *p* < 0.05). In contrast, the progeny RRRRCC, which shares the same number of R genomes as RRRRCC^1^, displayed a lower HEP frequency of 74.24%. Offspring plants with RRRCC genetic composition (2n = 43, 44, 45) showed varying HEP frequencies, with RRRCC^2^ having the highest frequency at 69.09%, followed by RRRCC^1^ at 61.67%, and RRRCC at 58.73%. Overall, the rate of homoeologous pairing in euploids was lower compared to aneuploids across all autoallopolyploid varieties. Aneuploids showed higher HEP rates compared to euploids, with trivalent pairings between R and C chromosomes being common. Bivalents between R and C chromosomes were rare, with only nine cells exhibiting this configuration for analysis. Quadrivalents of type IV^R-R-R-R^ were more likely in RRRRCC (2n = 54, 55) cells, while C chromosome cells exhibited quadrivalents in both euploids.

### 2.4. Chromosome Segregations in These Progenies

**Figure 6:**
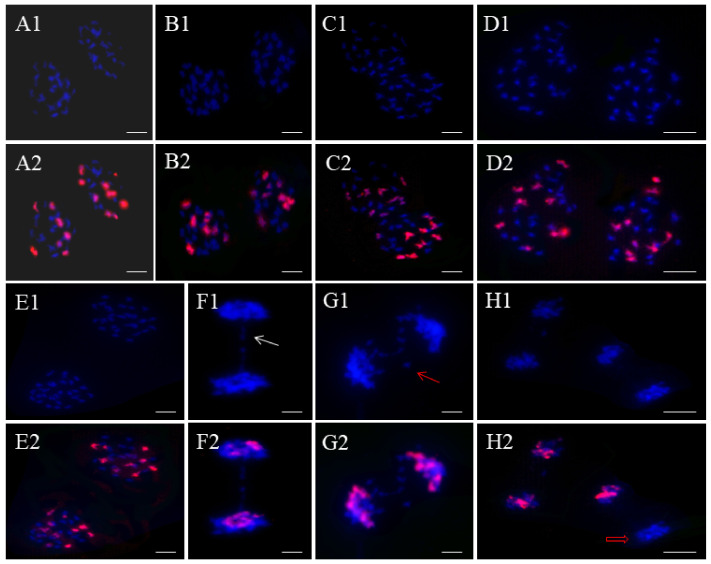
BAC-FISH analyses of chromosome behaviors of each offspring of RRRC at anaphase I (AI) and anaphase II (AII). The blue signal indicates DAPI staining, and red indicates labeled BAC BoB014O06. (**A1**,**A2**) PMC chromosomal configurations of RRRCC^2^ separated as 19 (12R + 7C): 24 (15R + 9C). (**B1**,**B2**) PMC chromosomal configurations of RRRCC^1^ separated as 25 (17R + 8C): 19 (10R + 9C). (**C1**,**C2**) PMC chromosomal configurations of RRRCC at AI. Chromosomes separated as 23 (14R + 9C): 22 (13R + 9C). (**D1**,**D2**) PMC chromosomal configurations of RRRRCC at AI. Chromosomes separated as 29 (20R + 9C): 25 (16R + 9C). (**E1**,**E2**) PMC chromosomal configurations of RRRRCC^1^ at AI separated as 28 (18R + 10C): 27 (18R + 9C). Irregularity of chromosome behavior occurs at AI and AII. (**F1**,**F2**) The formation of chromosome bridge occurs at AI from meiotic cell of RRRCC^1^ (white arrow). (**G1**,**G2**) Lagged chromosomes occur at AI from meiotic cell of RRRCC^2^ (red arrow). (**H1**,**H2**) None of the C chromosomes separate to the cluster (red hollow arrow). Bar, 10 μm.

In our study, we extensively analyzed the pollen mother cell (PMC) chromosome compositions of the offspring during anaphase I, as depicted in Figure 6. Among the progeny, except for RRRCC and RRRRCC, there was an uneven distribution of R and C chromosomes due to varying chromosome numbers. For instance, RRRCC^2^ (Figure 6(A1)) showed a more consistent separation of R and C chromosomes with a division ratio of 19 (12R + 7C): 24 (15R + 9C) compared to RRRCC^1^ (Figure 6(B1)) with a division ratio of 25 (17R + 8C): 19 (10R + 9C). While RRRCC exhibited an unequal distribution of R chromosomes, the two C subgenomes were usually distributed equally, as seen in the ratio of 23 (14R + 9C) to 22 (13R + 9C) in Figure 6(C1). In the case of RRRRCC, an autoallohexaploid, we observed an unexpected distribution with unequal R chromosomes but equal C chromosomes at a ratio of 29 (20R + 9C) to 25 (16R + 9C) (Figure 6(D1)). An interesting observation was made in RRRRCC1, which had an odd number of C chromosomes, resulting in a non-uniform distribution of C chromosomes but an even distribution of R chromosomes at a ratio of 28 (18R + 10C) to 27 (18R + 9C) (Figure 6(E1)). Additionally, irregular chromosome behavior was noted in two instances during anaphase I, where R chromosomes formed bridges and lagged behind (Figure 6(F1,G1)). A particularly intriguing scenario was observed in RRRRCC1 during anaphase II, where one side had an unreduced number of C chromosomes, while the other side only contained R chromosomes (Figure 6(H1)).

## 3. Discussion

The emergence of new species with multiple genomes from different species is common in plant evolution, but autoallopolyploids possessing chromosomes from both parental species are rare in nature [36,37]. This study employed fluorescence in situ hybridization to identify several autoallopolyploids resulting from unexpected self-fertilization of an allotetraploid (RRRC). The resulting offspring exhibited increased complexity and higher levels of polyploidy. The research further examined the phenotypes, chromosome pairing, and segregation of these progenies, providing crucial evidence for understanding the origin of autoallopolyploids and the relationship between the R and C genomes.

**Figure 7:**
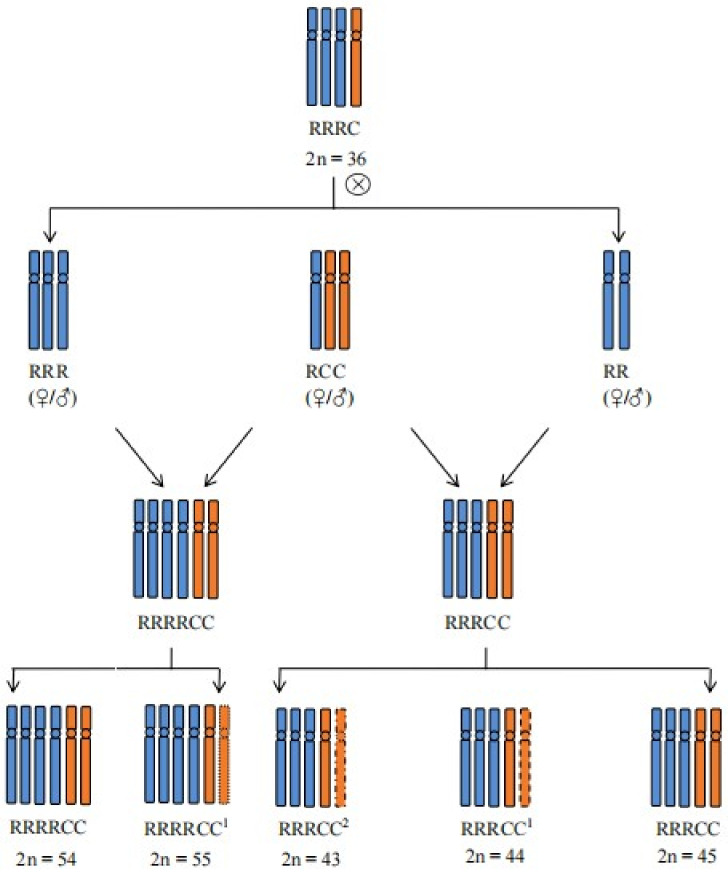
Imaginary representation of the origin of the autoallopolyploid offspring from RRRC hybrid. The diagrams with blue and red colors represent R and C genomes, respectively. The upper “1” and “2” indicate that the aneuploid plants have one or two chromosomes deviating from the chromosome number of euploid plants.

These offspring plants derived from the same parental plant displayed two identical characteristics in chromosome composition. Firstly, the C genome did not lose chromosomes; rather, it gained one or more genomes based on the original genome. Secondly, there was an unequal increase and decrease in the R and C chromosomes, with the R genome doubling. These characteristics resulted from various factors, categorizing the progenies into euploid and aneuploid groups, with chromosome numbers in both groups being very similar. This phenomenon is believed to be a result of the need for dosage compensation to maintain chromosome numbers close to euploid levels [38]. Statistical analysis indicated that the number of aneuploid plants among these offspring was lower than that of euploid plants, specifically RRRCC plants. Siegel et al. [39] also utilized the concept of dosage compensation to elucidate this phenomenon and proposed various explanations for the occurrence of aneuploidy, including SAC mutations, premature loss of chromatid cohesion, aberrant kinetochore attachments, supernumerary centrosomes, and meiotic segregation errors (missegregation during meiosis II and missegregation during meiosis I). In our study, we observed the unreduced single C genome at meiosis II in RRRRCC^1^, leading us to speculate that the increase in the C chromosome in these progenies resulted from the unreduced single C genome during gamete formation in RRRC. This observation led to the production of unreduced gametes, which included approximately three types: RRR, RCC, and RR, all of which met the criteria for unreduced gametes and are depicted in the schematic diagram (Figure 7). Additionally, we noted a lag in the R chromosome during meiosis I in these progenies, with no loss of R genome chromosomes. This observation led us to infer that all aneuploid R genome gametes generated by RRRC were nonviable during gamete formation, indicating that aneuploidy of the R chromosome resulted in lethal damage to gametes [5].

The distinct cytological behaviors of various genomes may be associated with their inherent characteristics and the genetic distance between relatives. In this study, we analyzed the pairing frequency of R and C chromosomes in each progeny at diakinesis and found that the homoeologous pairing frequency between these two genomes ranged from 58.73% to 78.33%. Furthermore, we compared the chromosome pairing frequency between R and C genomes in several hybrids including RRRC and RRC, as previously reported by Yu et al. [35]. A homoeologous pairing rate of 88.23% was reported in RRRC by Yu et al.; however, our study on the hybrid RRRCC (2n = 43, 44, 45), which had the same number of R genomes as RRRC, exhibited a pairing rate ranging from 58.73% to 69.09%. The apparent decrease in the pairing rate is likely attributable to the increase in the C genome, which corresponds to a reduction in the pairing rate. Intriguingly, the rate of homologous pairing in RRC exceeded that of RRRCC yet remained lower than that of RRRRCC. This indicates that the pairing frequency between R and C chromosomes is influenced by the number of R and C genomes; notably, a greater disparity between the number of C genomes and R genomes correlates with a higher pairing frequency. Cai et al. [17] generated five allohexaploid types of *Brassica* through interspecific hybridization, observing different homoeologous pairing frequencies. In autoallopolyploids, few univalents were formed, with euploids exhibiting almost none and aneuploids displaying slightly more frequent occurrences. The formation of bivalents between C and R was minimal, with homologous pairing being more prevalent than homoeologous pairing, thereby ensuring stable inheritance of one genome set. The coexistence of two genomes in multivalents at diakinesis enhances genetic diversity. A study by Zhou et al. [40] revealed that frequent homoeologous chromosome pairings between the A and C subgenomes contributed to the loss of chromosomes and chromosome segments within the *Brassica* species. Katche et al. [41] made a similar observation. Cai et al. [42] further proposed that one genome might possess a larger and more unstable size (C > R) compared to the other, leading to chromosome loss in the latter genome. Consequently, we speculate that the aneuploidy observed in this study may linked to the high frequency of homoeologous pairing and genome instability, resulting in the loss of C genome chromosomes. The autoallpolyploids obtained in this study not only reduce reproductive isolation between radish and *B. oleracea* [43] but also serve as intermediate materials for constructing both *B. oleracea* and radish characteristics.

Concentrating on agronomic traits facilitates comprehension of a species’ characteristics pertinent to suitable agricultural environments. The hybrids identified via fluorescence in situ hybridization in the present investigation exhibit enhanced attributes relative to grain weight, and their oil content may be evaluated in subsequent inquiries. The two parental plants mentioned in this study are edible vegetable crops, with radish boasting a growth cycle of six months and kale thriving within three to four months. The hybrids not only retain edibility but also display a truncated growth cycle of approximately four months compared to radish. Consequently, we think hybrids with such attributes may serve as intermediate materials in the future to improve radish germplasm resources, thereby significantly abbreviating the growth cycle of radish. Zhang et al. [44] also obtained a genetically stable intergeneric hybrid, *Raphanobrassica* (2n = 4x = 36), through intergeneric hybridization between radish (*Raphanus sativus*, 2n = 2x = 18) and kale (*Brassica oleracea* var. alboglabra, 2n = 2x = 18) with multiple generations of selective breeding. The progeny exhibited commendable agronomic properties, including resistance to clubroot disease and *Sclerotinia sclerotiorum*, indicating significant potential for breeding purposes. Consequently, a comparative analysis was conducted between Zhang’s and Yu’s research (Table 3). Despite the utilization of distinct varieties of materials, both resulted in the identical hybrid, *Raphanobrassica*, which was subsequently employed in their respective studies. Zhang et al. [44] elucidated the correlation between the morphological features and gene expression of the siliques of *Raphanobrassica*, thereby offering a reference for the investigation of gene expression patterns in remotely hybridized plants. Yu et al. obtained five hybrids through twice reciprocal crosses and investigated the pairing frequency of genomic chromosomes present in each hybrid. Subsequently, we identified five novel hybrids from the RRRC offspring.

## 4. Materials and Methods 

### 4.1. Chromosome Plant Materials and Cultivation Conditions

In total, 21 accessions and plants were used in this study, including two diploid parents, a *R. sativus* pure line called ‘Loutouqing’ and a *B. oleracea* pure line called ‘Chijielan’; an allotetraploid *Raphanobrassica* [34]; an autoallotetraploid plant (CCCR) derived from the interspecific hybridization between *Raphanobrassica* and *B. oleracea* (no offspring plants obtained); an autoallotetraploid plant (RRRC) derived from the interspecific hybridization between *Raphanobrassica* and *R. sativus* [33]; and 16 offspring plants from the autoallotetraploid. The seeds of the parental plants were provided by our laboratory. The allotetraploid *Raphanobrassica* was obtained through interspecific hybridization between the two parental plants and further whole-genome doubling. These materials were firstly cultured in pots (8 cm in length and width and 10 cm in depth) in an incubator under cultural conditions with a 16/8 h light/dark cycle, a temperature maintained at 22 °C, and a relative humidity level of 40%. When the plants grew to the two-leaf stage, these plants were then transplanted into larger pots (40 cm in diameter and 50 cm in depth) in the experimental field at Guizhou Normal University, Guiyang, China (26°23′11″ N, 106°38′32″ E), under the same cultural conditions as in the seedling stage. To obtain enough seeds, artificial supplementary pollination was carried out on these materials. To obtain the offspring seedlings of autoallotetraploid, the embryo rescue method was employed as described by Cui et al. [45]. Following a 20-day period of pollination, the immature embryos were cultivated on a Murashige and Skoog (MS) medium to obtain the offspring seedlings of autoallotetraploid. Then, these offspring plants were cloned in MS medium with 1.5 mg/L 6-benzylamine adenine (6-BA) and 0.25 mg/L naphthalene acetic acid (NAA) to generate enough plantlets for cytological and morphological studies [45].

### 4.2. Analysis of Cytology and Pollen Viability

In order to determine the chromosome number and chromosome configuration of the offspring plants, the ovaries from young flower buds were collected and treated with 8-hydroxyquinoline (2 mM) solution for 2.5 h at 22 °C in an incubator in the dark [17]. It is crucial to monitor the weather and temperature during this process, preferably on a sunny day with a temperature of approximately 20 °C. Then, these ovaries were treated with Carnoy’s solution, which was composed of ethanol and glacial acetic acid, with a volume ratio of 3:1 and stored at −20 °C for the determination of chromosome number and chromosome configuration. To check the chromosome behavior of these offspring plants, enough unopened buds were gathered and immediately treated with Carnoy’s solutions. The Carnoy’s solutions were changed every 6 h until the flower buds were completely discolored. The pollen fertility of these offspring plants depends on pollen stainability with 1% acetocarmine solution. In brief, at least three just blooming flowers from the main inflorescence were collected on a sunny day. More than 300 pollens per offspring plant were determined using 1% acetocarmine staining. The pollen germination test also was used to check the ability of these hybrids for pollen germination. The pollen grains were cultured on a solid medium composed of 1% agar, 10% sucrose, 0.01% boric acid, and 0.03% calcium nitrate. The pH of the medium was adjusted to 6.5 prior to the experiment. Following the culturing of the pollen onto the medium, the culture condition was maintained at a temperature of 25 °C, with 100% atmospheric humidity and in the absence of light, for a duration of 6 h. Cytological images were obtained using a computer-assisted microscope with a CCD camera (E200, Nikon, Tokyo, Japan).

### 4.3. Probe Preparation and Fluorescence In Situ Hybridization 

To detect the genome configuration and chromosome behavior of these offspring plants, fluorescence in situ hybridization (FISH) using C-genome-specific repeats as probes was carried out. The plasmid DNA of BAC BoB014O06 specific for the C-genome of *Brassica* species (provided by professor Zaiyun Li, Huazhong Agriculture University, Wuhan, China) giving rise a GISH-like pattern [39] was labeled with biotin-11-dCTP by random priming using the BioPrime DNA Labeling System kit (Invitrogen, Life Technologies, Carlsbad, CA, USA) according to the manufacturer’s protocol (Invitrogen, Life Technologies). The method of glass slide preparation was consistent with the work of Zhong et al. [45,46]. The slides were pre-treated with 500 ng/μL pepsin in 10 mM HCl for a duration of 20 min, followed by incubation with 10 mg/mL RNase in 2 × SSC for half an hour at 37 °C. Subsequently, they were fixed with 4% paraformaldehyde for 10 min, dehydrated using 75% and 100% ethanol for three minutes each, and finally air-dried. The hybridization mixture was composed of 50% deionized formamide, 2 × SSC, 10% dextran sulfate, 0.5% SDS, and 3 μL probes for each slide. This mixture was denatured at 85 °C for 10 min. Co-denaturation of the probes and chromosomal DNA on the slides was conducted at 85 °C for 7–8 min in a thermal cycler, followed by hybridization overnight at 37 °C in a humid chamber. Stringent washing was performed for 10 min in 0.1 × SSC with 20% deionized formamide at 40 °C. The immunodetection of biotinylated and digoxigenated DNA probes was carried out utilizing Cy3-labeled streptavidin (KPL, St. Louis, MO, USA) and anti-digoxigenin conjugate-FITC (Roche, Basel, Switzerland), respectively. Ultimately, the preparations were counterstained with 4′-6-diamidino-2-phenylindole (DAPI) solution (Roche, Basel, Switzerland) at a concentration of 1 mg/mL and mounted in an antifade oil (Vector Laboratories, Peterborough, UK).

The pictures of FISH analysis were captured using a fluorescence microscope (N80i, Nikon, Japan) with a CCD camera and then merged using Adobe Photoshop (version 7.0) software.

### 4.4. Phenotype Evaluation of These Offspring Plants 

The morphological characteristics of offspring plants, including assessments of the leaf, stem, root and flower color, leaf margin serration, leaf lobe quantity and structure, leaf and stem hairiness, and growth habit, were comprehensively evaluated [47]. The morphology and dimensions of fully expanded leaves from the same location on these offspring, as well as from two controlled parents, were examined. Attention must be paid to the presence of hairiness on both the leaves and stems during both the seedling stage and the adult seedling stage.

### 4.5. Data Statistics Processing and Graphing 

The one-way analysis of variance (ANOVA) and Chi-square test were used to determine the significance between two comparisons with a cutoff value of *p* < 0.05. A significant difference among multiple comparisons was determined using Tukey’s HSD (*p* < 0.05). The box plot of pollen viability comparisons was made using GraphPad Prism5.

## 5. Conclusions

This study identified two main genome configurations in these offspring plants, RRRCC (2n = 43, 44, 45) and RRRRCC (2n = 54, 55), which were reported for the first time, to the best of our best knowledge, through fluorescence in situ hybridization in the progeny of tetraploid-RRRC derived from interspecific hybridization between *Raphanobrassica* and radish. These offspring exhibited higher ploidy levels and more complex characteristics compared to their parents, with an increased number of branches and seed weight. Significant declines in fertility were observed as the proportion of R and C genome chromosomes increased unexpectedly. Subsequently, it was discovered that the frequency of R and C genome pairing varied among different progenies at diakinesis and anaphase II. This observation elucidated the formation of these offspring plants, highlighting the presence of an unreduced single C genome for the first time. These hybrids obtained in this study reduce reproductive isolation between two distinct species and have great potential as intermediate materials for further analysis in *Brassica* in the long run.

## Figures and Tables

**Table 1:** Summary of chromosome composition of the offspring plants from RRRC hybrid.

Plant Types	Total Chromosome Number	Genome Configuration	Number of Plants
RRRCC^2^	43	27R + 16C	4
RRRCC^1^	44	27R + 17C	5
RRRCC	45	27R + 18C	5
RRRRCC	54	36R + 18C	1
RRRRCC^1^	55	36R + 19C	1
**Total**	-	-	16

**Table 2:** Summary of HEP incidents in PMCs of the offspring.

Plant Types	Total PMCs	Ratio	Allosyndesis Types	Autosyndesis Types
RRRCC^2 (c)^	55	69.09%	R-C; R-R-C; C-C-R; R-C-R; C-R-C; R-C-C-R;	C-C; C-C-C; R-R; R-R-R;
RRRCC^1 (d)^	60	61.67%	R-C; R-R-C; C-C-R; R-C-R; C-R-C; R-C-C-R; C-C-C-R; C-C-R-R-R-C;	C-C; C-C-C; R-R; R-R-R; R-R-R-R;
RRRCC ^(e)^	63	58.73%	R-C; R-R-C; C-C-R; R-C-R; C-R-C; R-C-C-R; C-C-C-R; R-R-C-C-C-C;	C-C; C-C-C; C-C-C-C; R-R; R-R-R; R-R-R-R;
RRRRCC ^(b)^	66	74.24%	R-C; R-R-C; C-C-R; R-C-R; C-R-C; R-C-C-R; C-C-C-C-R; R-R-R-R-R-C;	C-C; C-C-C; C-C-C-C; R-R; R-R-R; R-R-R-R;
RRRRCC^1 (a)^	60	78.33%	R-C; R-R-C; C-C-R; R-C-R; C-R-C; R-C-C-R; C-C-C-R; C-C-C-R-R;	C-C; C-C-C; R-R; R-R-R; R-R-R-R;

Shared letters a–e are extremely significantly different as detected by Chi-seq, *p* < 0.

**Table 3:** The comparison in the three studies.

Study	Materials	Hybrid Types	Results
“Genome-wide unbalanced expression bias and expression level dominance toward *Brassica* oleracea in artificially synthesized intergeneric hybrids of Raphanobrassica” by Zhang et al. [44]	*Raphanus sativus* *Brassica oleracea* var. alboglabra Raphanobrassica	*Raphanobrassica* (2n = 36)	Transcriptome shock was observed in five tissues of Raphanobrassica. Genome-wide unbalanced biased expression and expression level dominance were also discovered, and both of them were toward *B. oleracea* in *Raphanobrassica*, which is consistent with the observed phenotypes.
“Identification, Characterization, and Cytological Analysis of Several Unexpected Hybrids Derived from Reciprocal Crosses between *Raphanobrassica* and Its Diploid Parents” by Yu et al. [35]	*Raphanus sativus* var. Loutouqing *Brassica oleracea* var. Chijielan *Raphanobrassica*	Several hybrids including RRC (2n = 27), CCR (2n = 27), RRRC (2n = 36), CCCR (2n = 36), and RRC’ (2n = 26)	Two expected hybrids (RRC and CCR) and three unexpected hybrids (RRRC, CCCR, and RRC’) were reported for the first time, and the study demonstrated that the unexpected hybrids exhibited significantly more intergenomic chromosome pairings than the expected hybrids.
Characteristics and Cytological Analysis of Several Novel Allopolyploids and Aneuploids between *Brassica oleracea* and *Raphanus sativus* by the authors of the present study	*Raphanus sativus* var. Loutouqing *Brassica oleracea* var. Chijielan Two hybrids RRRC and CCCR derived from Yu’s study	Two allopolyploids including RRRCC (2n = 45) and RRRRCC (2n = 54). Three anuepolyploids including RRRCC^2^ (2n = 43), RRRCC^1^ (2n = 44), and RRRRCC^1^ (2n = 55)	Two main genome configurations in these offspring plants, RRRCC (2n = 43, 44, 45) and RRRRCC (2n = 54, 55), were identified for the first time. It was discovered that the frequency of R and C genome pairing varied among different progenies at diakinesis and anaphase II. This observation elucidated the formation of these offspring plants, highlighting the presence of an unreduced single C genome for the first time.

## Data Availability

Data is contained within the article or Supplementary Material.

## Supplementary Materials

The following supporting information can be downloaded at https://www.mdpi.com/article/10.3390/ijms25158368/s1.
