# Supplementary material for: Characteristics and Cytological Analysis of Several Novel Allopolyploids and Aneuploids between Brassica oleracea and Raphanus sativus
Source: Int J Mol Sci. 2024 Jul 31;25(15):8368. doi: 10.3390/ijms25158368 (PMC11313488; doi:10.3390/ijms25158368)
Supplement: Supplementary file 1 [file ijms-25-08368-s001.zip › ijms-3108809-supplementary.pdf]

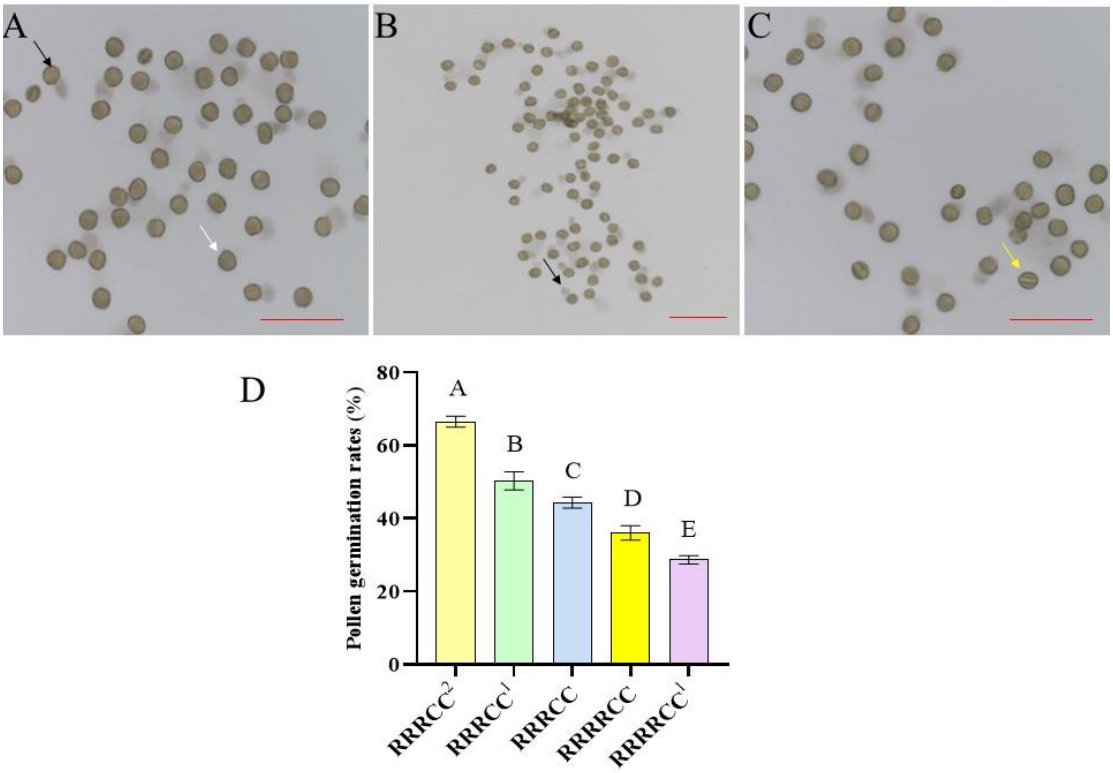

Figure S1. Pollen germination analysis of the offspring of RRRC. (**A–C**) The pollen characteristics of RRRCC1, RRRCC and RRRRCC. The black arrows indicate the germination of pollen grains. White arrow indicates ungerminated pollen grains. The yellow arrow points to pollen grains where pollen pore is visible. Bar: 100 µm. (**D**) Comparison of pollen germination rates across above-mentioned hybrids. Shared letters A-E extremely significantly different detected by One-way analysis (ANOVA), $p < 0.05$.
